# Improving Bone Formation by Guided Bone Regeneration Using a Collagen Membrane with rhBMP-2: A Novel Concept

**DOI:** 10.3390/jfb14030170

**Published:** 2023-03-22

**Authors:** Narae Jung, Jaehan Park, Sang-Hyun Park, Seunghan Oh, Sungtae Kim, Sung-Won Cho, Jong-Eun Kim, Hong Seok Moon, Young-Bum Park

**Affiliations:** 1 Department of Clinical Dentistry, Oral Science Research Center, BK21 FOUR Project, College of Dentistry, Yonsei University, 50-1 Yonsei-ro, Seodaemun-gu, Seoul 03722, Republic of Korea; jnrgood1217@yuhs.ac; 2 Department of Prosthodontics, College of Dentistry, Yonsei University, 50-1 Yonsei-ro, Seodaemun-gu, Seoul 03722, Republic of Korea; imfunny1106@gmail.com (J.P.);; 3 Osong Research Institute, TaeWoong Medical Co., Ltd., 55-7 Osongsaengmyeong 2-ro, Heungdeok-gu, Cheongju 28161, Republic of Korea; 4 Department of Dental Biomaterials and Institute of Biomaterials & Implant, College of Dentistry, Wonkwang University, 460 Iksandae-ro, Iksan 54538, Republic of Korea; 5 Department of Periodontology, Dental Research Institute, School of Dentistry, Seoul National University, 101 Daehak-ro, Jongno-gu, Seoul 03080, Republic of Korea; 6 Division of Anatomy and Developmental Biology, Department of Oral Biology, College of Dentistry, Yonsei University, 50-1 Yonsei-ro, Seodaemun-gu, Seoul 03722, Republic of Korea

**Keywords:** guided bone regeneration, rhBMP-2, collagen membrane, biphasic calcium phosphate, bone formation

## Abstract

We examined whether recombinant human bone morphogenetic protein-2 (rhBMP-2) when applied to collagen membranes, would reinforce them during guided bone regeneration. Four critical cranial bone defects were created and treated in 30 New Zealand white rabbits, including a control group, critical defect only; group 1, collagen membrane only; group 2, biphasic calcium phosphate (BCP) only; group 3, collagen membrane + BCP; group 4, collagen membrane with rhBMP-2 (1.0 mg/mL); group 5, collagen membrane with rhBMP-2 (0.5 mg/mL); group 6, collagen membrane with rhBMP-2 (1.0 mg/mL) + BCP; and group 7, collagen membrane with rhBMP-2 (0.5 mg/mL) + BCP. After a 2-, 4-, or 8-week healing period, the animals were sacrificed. The combination of collagen membranes with rhBMP-2 and BCP yielded significantly higher bone formation rates compared to the other groups (control group and groups 1–5 < groups 6 and 7; *p <* 0.05). A 2-week healing period yielded significantly lower bone formation than that at 4 and 8 weeks (2 < 4 = 8 weeks; *p <* 0.05). This study proposes a novel GBR concept in which rhBMP-2 is applied to collagen membranes outside instead of inside the grafted area, thereby inducing quantitatively and qualitatively enhanced bone regeneration in critical bone defects.

## 1. Introduction

Guided bone regeneration (GBR), which was developed on the basis of the principles of guided tissue regeneration, is the most widely used method for reinforcing bone in local alveolar defects [1,2,3,4]. Membranes are essential elements of GBR because they prevent the invasion of fast-growing epithelial and connective tissues into the defect site, thereby facilitating the formation of slow-growing bone tissue [5]. The membranes used initially were non-absorbable, which were subsequently replaced by absorbable membranes [6,7]. Collagen membranes are widely used in clinical practice and are sometimes used in conjunction with bone grafting inside the defect. Biphasic calcium phosphate (BCP) is a widely used synthetic bone graft material. It is composed of a mixture of hydroxyapatite and β-tricalcium phosphate and has large pores, which allow it to form a strong link with collagen membranes [8,9]. Therefore, BCP is used for bone grafting in sinus grafts and dental implants [10].

The degree of bone formation varies during bone grafting, depending on the condition of the osteogenic cells at the graft site, the surface and internal structure of the bone graft material, and cell affinity [11]. Various growth factors have been developed to optimize these aspects and promote bone formation. Collagen membranes can continuously release growth factors for a certain period, with ideal release kinetics for growth factors [12]. The use of growth factors and collagen membranes accelerates GBR [13]. Recombinant human bone morphogenetic protein-2 (rhBMP-2) and rhBMP-7 facilitate the differentiation of osteoblasts and induce osteoinduction [14,15]. rhBMP-2 is mainly used for osteoinduction in the oral and maxillofacial region [16,17]. It induces bone regeneration when applied to bone graft materials [18,19,20]. The carrier increases the osteogenesis-inducing and regenerative abilities of rhBMP-2, and its regeneration potential depends on the carrier system [21,22,23]. The clinical complications associated with the use of rhBMP-2 include tumor growth, ectopic bone formation, osteolysis, urinary system problems, bone cysts, and inflammation [24]. Lee et al. reported that the use of rhBMP-2 inside a defect increased the adipocyte count and cystic changes after healing, decreasing the stability of the graft site and the quality of the new bone [25]. Therefore, bone-formation studies using rhBMP-2 have recommended methods that limit adverse effects, such as the use of vascular endothelial growth factor, the simultaneous application of a basic fibroblast growth factor, and the use of other carrier systems [26,27,28].

**Figure 1:**
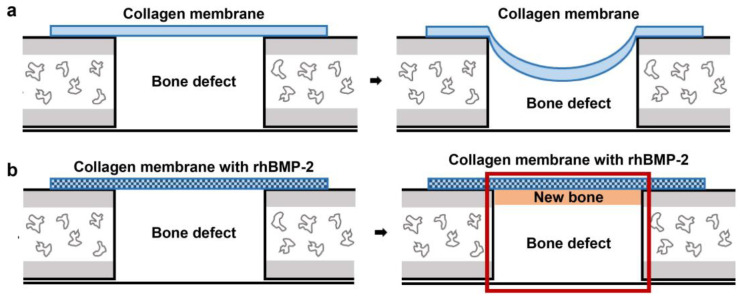
Schematic diagram of the original (**a**) vs. novel concept (**b**) of GBR (**a**) Disadvantages of the original GBR concept for critical cranial bone defect, (**b**) Novel concept of GBR using collagen membrane with rhBMP-2.

This study examined the effect of the application of rhBMP-2 with a collagen membrane for bone regeneration in critical cranial bone defects. Absorbent membranes gradually collapse during GBR and provide poor space maintenance. Since the existing GBR methods focus on the inside of the defects [29,30], this study examined the effect of reinforcing the membrane from the outside (Figure 1).

The collagen membrane continuously releases rhBMP-2, which increases alkaline phosphatase and osteocalcin (bone gamma-carboxyglutamate protein) levels, consequently enhancing bone regeneration activity [31]. The release of rhBMP-2 is more rapid during the early stages of healing. Thus, this combination is suitable for space maintenance and isolation [32].

The null hypothesis of this study was that the addition of rhBMP-2 to collagen membranes outside the defect would produce no difference in new bone formation during GBR in comparison with that observed by collagen membranes alone. This study aimed to determine whether the combination of a collagen membrane and rhBMP-2 outside the defect would produce a more favorable environment for bone regeneration inside the bone defect, the isolation of the defect, and the promotion of long-term bone formation. We proposed a novel concept for GBR using a collagen membrane with rhBMP-2 outside the defect instead of in the grafted area.

## 2. Materials and Methods

### 2.1. Animals and Materials

#### 2.1.1. Experimental Animals

Thirty 16–20-week-old New Zealand white laboratory rabbits weighing 3–3.5 kg were used in this study (weight was measured at the beginning of the week). The animals were quarantined, acclimatized for one week, and fed a standard laboratory diet. All experimental procedures (such as breeding, management, and surgical procedures) which were used in this study were approved by the Animal Care and Use Committee of Yonsei Medical Center, Seoul, Korea (approval no. 2016-0062). The Animal Experimental Ethics Committee of Yonsei Medical Center ensured that the animal experiments were conducted scientifically and ethically according to the use and management program of laboratory animals, based on the Guide for the Care and Use of Laboratory Animals (National Research Council, USA). Experiments involving live animals were performed in accordance with the ARRIVE guidelines.

#### 2.1.2. Collagen Membrane

This study employed highly pure, type-I, 300-μm-thick collagen membranes derived from bovine tendons (Genoss, Suwon, Republic of Korea), which were designed to last for more than six months when applied to the human body. They had no pores on the upper or lower surfaces and had a dense multilayer structure.

#### 2.1.3. Biphasic Calcium Phosphate

Osteon II (Genoss, Suwon, Republic of Korea), a type of BCP with hydroxyapatite: β-tricalcium phosphate ratio of 30:70, 250-μm pores, a porosity of 70%, and particle sizes of 0.5–1.0 mm was used in this study. The appropriate dose for 8-mm-diameter bone defects in rabbit calvaria is 70 mg, which was used for each bone defect.

#### 2.1.4. rhBMP-2

*Escherichia coli*-derived rhBMP-2 (GENOSS, Suwon, Republic of Korea) was used to prepare BMP-2. In clinical practice, rhBMP-2 is used at concentrations of less than 1.5 mg/mL [33]. Chung et al. studied new bone formation using concentrations of 0.5–1.0 mg/mL [18]. Thus, rhBMP-2 concentrations of 0.5 and 1.0 mg/mL were used in this study. rhBMP-2 solutions with concentrations of 0.5 and 1.0 mg/mL were prepared by diluting 25 or 50 μg of rhBMP-2 in 50 μL of saline. The control group received only 50 μL of physiological saline.

### 2.2. Study Design

#### 2.2.1. Groups

The rabbits were divided into eight groups: the control group did not receive any transplants; group 1 received only collagen membranes; group 2 received only BCP; group 3 received both collagen membranes and BCP; groups 4 and 5 received collagen membranes with 1.0, and 0.5 mg/mL of rhBMP-2, respectively; and groups 6 and 7 received BCP and collagen membranes with 0.5 and 1.0 mg/mL of rhBMP-2, respectively.

**Figure 2:**
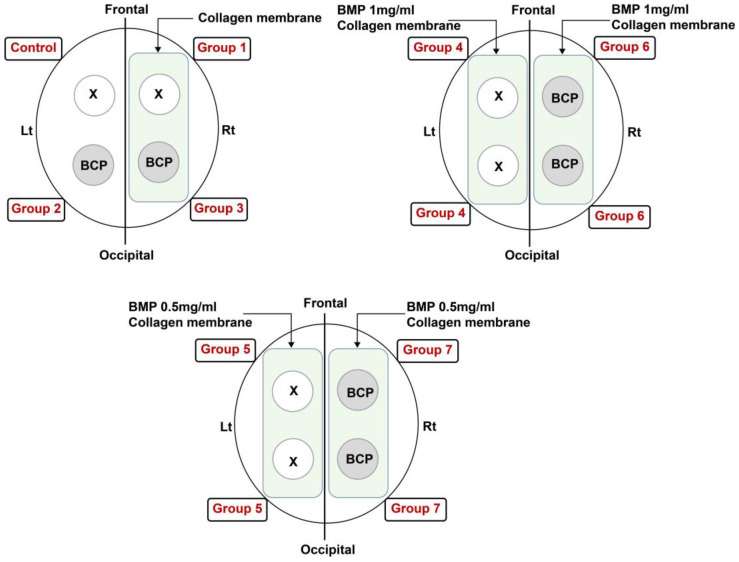
Schematic diagram of surgery and group distribution.

Four types of bone defects were created in the skull of each animal, and each of the four bone defects of each individual was assigned to a different group to reduce individual specificity. However, to prevent interference during the surgical procedure, only one rhBMP-2 concentration group was used per rabbit (Figure 2). Thus, 12 rabbits were included in the control group and groups 1, 2, and 3; nine rabbits were included in groups 4 and 6; and nine rabbits were included in groups 5 and 7. The animals were divided evenly into three groups and allowed to heal for 2, 4, or 8 weeks.

#### 2.2.2. Surgical Protocol

The rabbits were anesthetized using the subcutaneous injection of zolazepam (1.5 mg/kg; Zoletil; Virbac Korea Co., Seoul, Republic of Korea) and the intramuscular injection of xylazine HCl (5 mg/kg; Rompun; Bayer Korea Co., Seoul, Republic of Korea). After sterilizing the skin of the skull with povidone-iodine, local anesthesia was induced with 2% lidocaine (lidocaine HCl; Huons, Seongnam, Republic of Korea) containing 1:80,000 epinephrine. The defects created on the exposed skull surface were circular, with a depth of 1–2 mm and an outer diameter of 8 mm, which extended up to the inferior cortical bone without damaging the dura mater. rhBMP-2 dilutions, 10 × 20 mm^2^ collagen membranes, and physiological saline were prepared as appropriate for each group. The collagen membranes were soaked in 50 μL of rhBMP-2 solution for 30 min, as appropriate for each group. The collagen membranes for groups 1 and 3 were soaked in 50 μL of physiological saline. BCP was transplanted into each bone defect, as appropriate, and the collagen membranes were placed over the bone defects and fixed with micro pins. The periosteum of the skull was sutured first with 4–0 vicryl absorbable sutures (Ethicon, Somerville, NJ, USA), followed by the closure of the epidermis with sutures.

#### 2.2.3. Sacrifice

The animals were allowed to heal for periods of 2, 4, or 8 weeks, after which they were euthanized using the intravenous injection of concentrated sodium pentobarbital. The epidermis was incised, and the periosteum was isolated. Specimens were obtained by removing the marginal bone using a handpiece while maintaining a sufficient distance from the bone defects. The front sides of the specimens were marked.

#### 2.2.4. Histological and Histomorphometric Analysis

The specimens were fixed in 10% formalin for six weeks before being washed and demineralized in 2.5% NaOCl and 17% EDTA solution for 18 days. After embedding, the specimens were sliced to create sections of 3–4 μm thickness. The slices were then cut, along the sagittal plane, passing through the center of the bone defect in the anterior and posterior directions. The specimens were subjected to hematoxylin-eosin (H and E), Russell–Movat pentachrome staining (American MasterTech, USA), and tissue slides were prepared. The specimens were photographed at 12.5× and 40× magnifications using a BX50 optical microscope (Olympus, Tokyo, Japan). The new bone, remaining bone graft material (RB), fibrovascular connective tissue (FCT), and shrinkage volume (SV) (which is the unfilled volume of the defect) were measured within the defect area using Image-Pro Plus software (Media Cybernetics, Silver Spring, MD, USA). Each area was described as a percentage of the total defect area.

#### 2.2.5. Immunohistochemical Analysis

Immunohistochemical staining was performed with an anti-osteocalcin antibody (OCG3) to determine the vascularity and density of the osteoblasts. Anti-osteocalcin antibody (Abcam; ab13420, 1/150) and anti-mouse horseradish peroxidase (HRP; Abcam, ab205719, 1/150) were used according to the manufacturer’s instructions.

### 2.3. Statistical Analysis

The histological data were subjected to statistical analysis. Normality was confirmed using the Shapiro–Wilk test. Data were presented as the mean and standard deviation. The two-way ANOVA was performed to determine whether the rate of bone formation differed according to the healing period for each group. A postmortem analysis [least significant difference (LSD) method] was performed to determine the presence of any differences in the treatment groups. SPSS version 25.0 (IBM Corp., Armonk, NY, USA) was used for statistical calculations. Statistical significance was set at 5%, and *p*-values < 0.05 were considered statistically significant.

## 3. Results

### 3.1. Histological Findings (H and E Staining)

**Figure 3:**
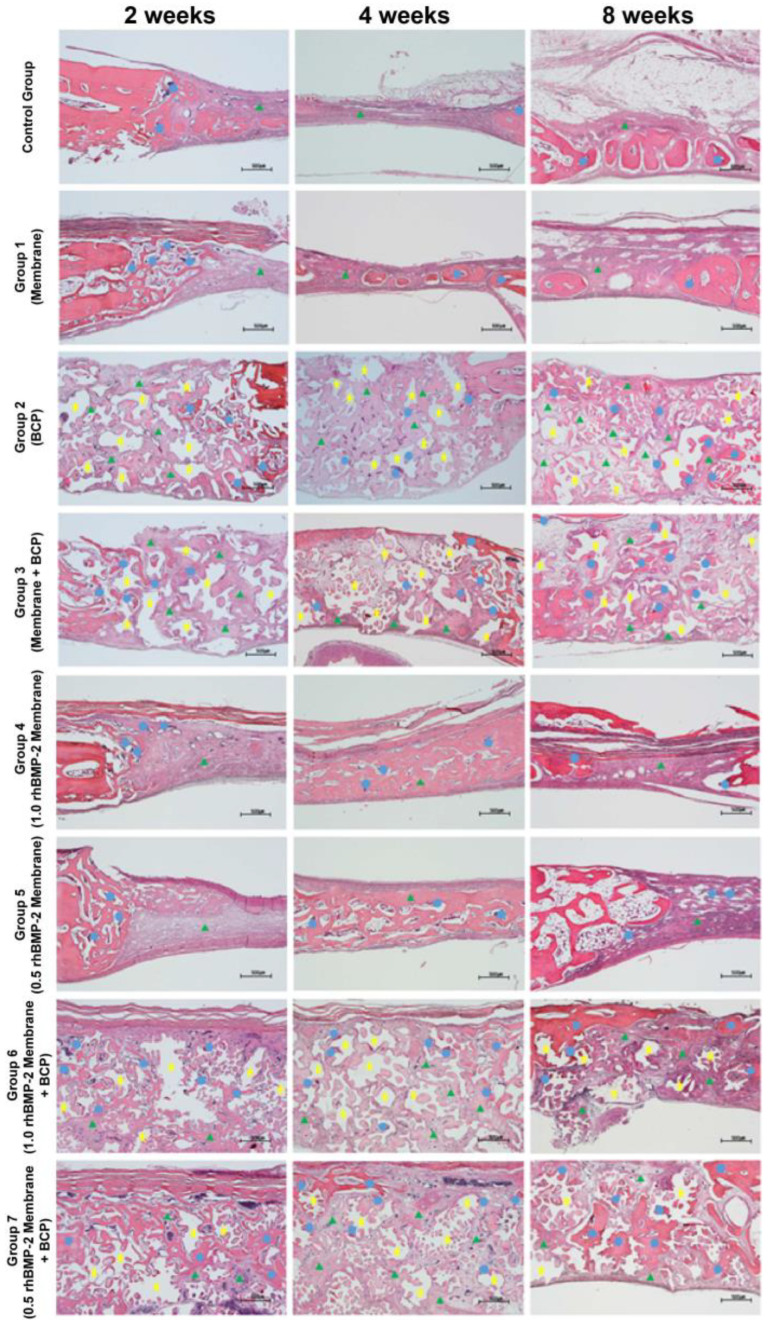
Histological specimens for each group after healing periods of 2, 4, and 8 weeks. Blue circles, new bone; yellow stars, remaining bone graft material; green triangles, connective tissue Scale bar: 500 µm.

Figure 3 shows images of the healing process in H and E-stained tissue specimens from the control and experimental groups. At 2 weeks, new bone (blue circles) formed only near the defect boundary in the control group, most of which was woven bone derived from the vicinity of the existing bone. In groups 2, 3, 6, and 7 that received BCP, new bones (blue circles) started to form around the RB (yellow stars) from the boundaries of the existing left and right bones. After 4 weeks of healing, the experimental groups treated with collagen membrane and BCP showed new bone (blue circles) formation at the center of the defect. The control group showed more adipose tissue on the outer side of the superior defect than after 2 weeks. After 8 weeks of healing, the upper periosteum of the control group was completely covered with a layer of adipose tissue, but there was little new bone. In group 3, new bone (blue circles) formed on the upper part connected to the existing bone, and loose connective tissue (green triangles) formed around the RB (yellow stars). In groups 6 and 7, new bone (blue circles) covered the upper part of the defect continuously, and the specimens showed a significant decrease in the RB (yellow stars) without adipose tissue formation. In most experimental groups, the more new, histologically mature bone had formed in the center of the defects, and the boundaries between the new and existing bone became less clear (Figure 3).

### 3.2. Histomorphometric Findings

Three of the 120 specimens were damaged during sample extraction. Of the 117 specimens, 17 were excluded as outliers, and the remaining 100 were included in the analysis.

### 3.3. New Bone Formation

**Figure 4:**
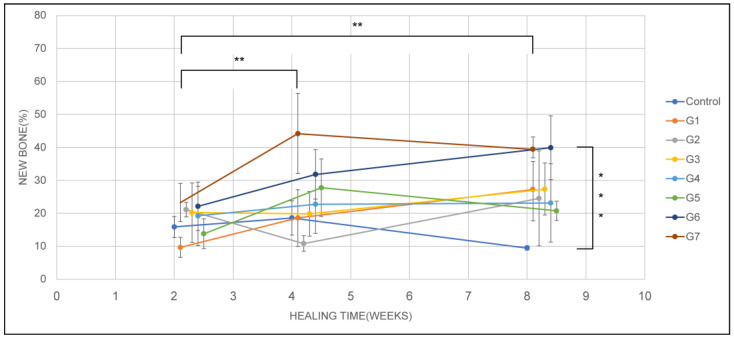
New bone formation by healing period and group. Statistically significant (*, *p* < 0.01; **, *p* < 0.001; ANOVA test).

A two-way ANOVA was conducted to determine the differences in bone formation rates according to the groups and healing periods. The bone formation rate was the lowest in the control group and highest in group 7. The *p*-values according to the groups and healing period were < 0.001 and 0.008, both of which were less than 0.05 (Table 1, Figure 4). A post hoc analysis using the LSD method confirmed that the bone formation rates in the control group and groups 1, 2, 3, 4, and 5 were lower than those in groups 6 and 7. The 2-, 4-, and 8-week healing periods showed average bone formation rates of 18.71%, 23.29%, and 27.03%, respectively. Postmortem analysis using the LSD method showed that the bone formation rate after a healing period of 2 weeks was lower than that after 4 and 8 weeks.

Groups 6 and 7 showed significantly greater new bone formation at 4 and 8 weeks compared with the other groups.

### 3.4. Histological Components

**Figure 5:**
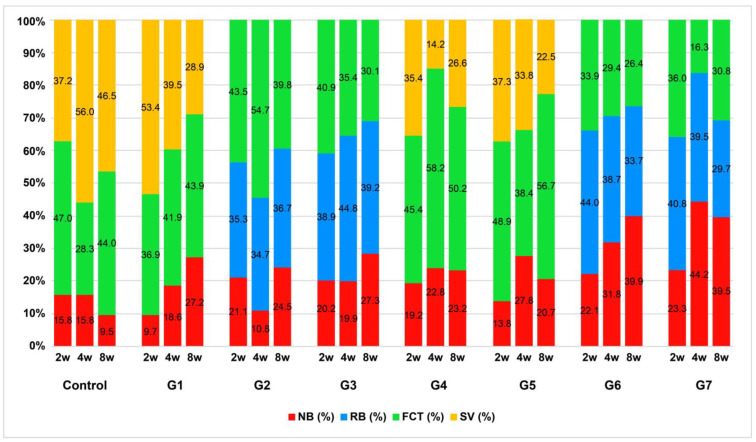
Cumulative graph of the histologic components in each group. As the healing period increased, the new bone (%) in groups 6 and 7 increased significantly, while the RB (%) and FCT (%) showed a relative decline.

New bone (%), RB (%), FCT (%), and SV (%) were measured. Each area was converted into a percentage of the total area of the defect. Figure 5 shows a graph of the patterns of changes in the four parameters across the healing period in each group. In all groups, except the control, new bone was greater at 8 weeks than at 2 weeks. Groups 6 and 7 showed a significantly greater improvement in new bone than that in the other groups, while the RB and FCT decreased over time. The SV of the groups that did not receive BCP was lower in groups 4 and 5 than in the control group and group 1 (Figure 5).

### 3.5. Histological Findings (Russell-Movat Pentachrome Staining)

**Figure 6:**
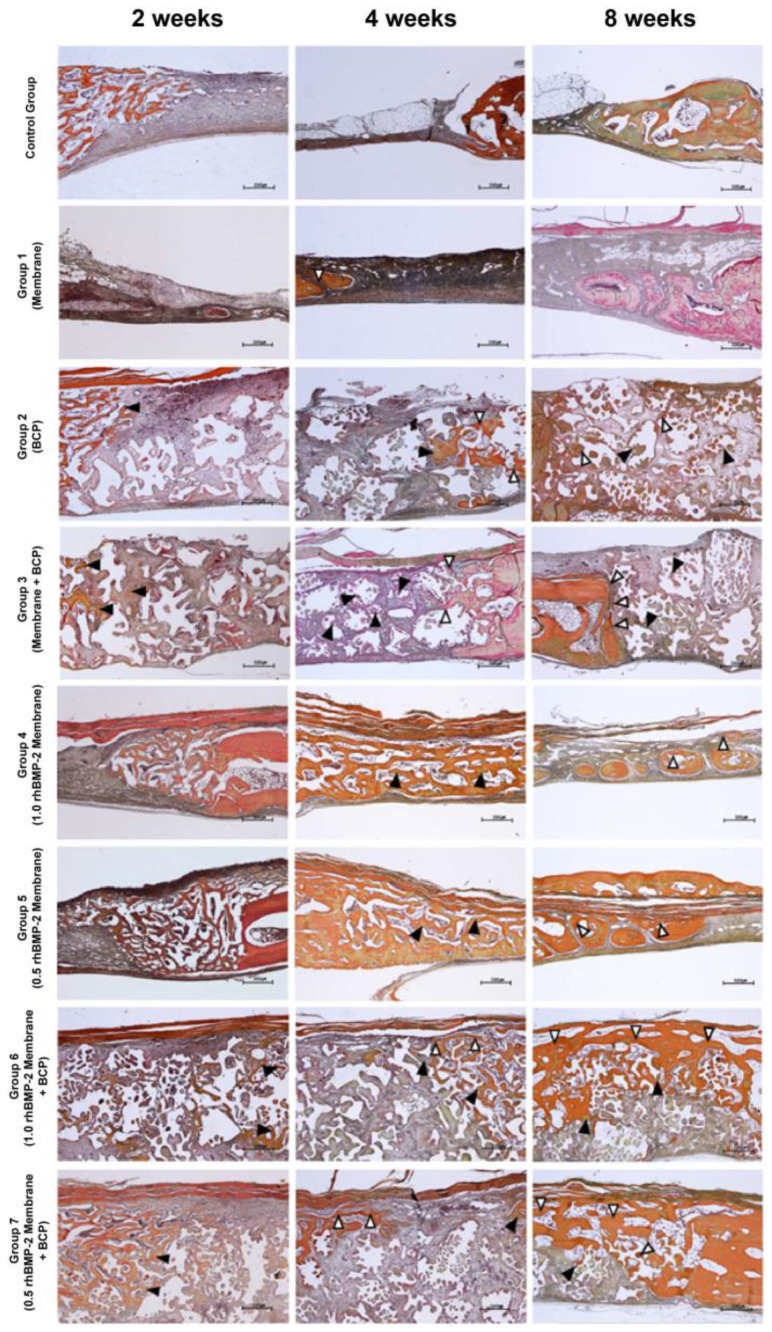
Histological specimens for each group after 2-, 4-, and 8-week healing periods (Pentachrome staining). Black arrowhead, immature new bone; white arrowhead, mature new bone. Scale bar: 500 µm.

Figure 6 shows images of pentachrome-stained tissue specimens of the control and experimental groups for visualizing the maturity of new bone. Mature bone was stained yellow to red, whereas immature bone was stained green. At 2 weeks, most groups showed a clear boundary between the existing and new bone (black arrowheads) on both sides. In groups 2, 3, 6, and 7, all of which received BCP, new bone (black arrowheads) formed near the existing bone, and collagen fibers formed around the bone graft material. In groups 6 and 7, new bone (black arrowheads) grew from the lower part of the membrane to the center of the defect. However, this new bone was immature, and many collagen fibers were formed in the upper part and around the bone graft material. After 4 weeks of healing, group 3 showed the formation of mature new bone (white arrowheads) around the existing bone, while new immature bone (black arrowheads) was only found inside the bone graft in the defect. In groups 4 and 5, new immature bone (black arrowheads) formed even at the center of the defects. In groups 6 and 7, mature new bone (white arrowheads) was observed in the upper part, which was not fully connected. In most groups, the new bone near the defect boundary and upper side were thicker and more mature than that at 2 weeks, and the boundary between the new and existing bone was relatively clear. After 8 weeks of healing, all groups showed new bone that was histologically more mature than in the earlier period, and the boundary between the new and existing bone was unclear. In groups 2 and 3, mature new bone (white arrowheads) was observed around the existing bone, but inside the defects, new bone (black arrowheads) and collagen fibers only formed around the bone graft material. In groups 4 and 5, some mature new bone (white arrowheads) formed at the center of the defect. In groups 6 and 7, continuous mature new bone (white arrowheads) was connected to the upper part of the defect.

### 3.6. Immunohistochemical Findings (Anti-Osteocalcin Antibody Staining)

**Figure 7:**
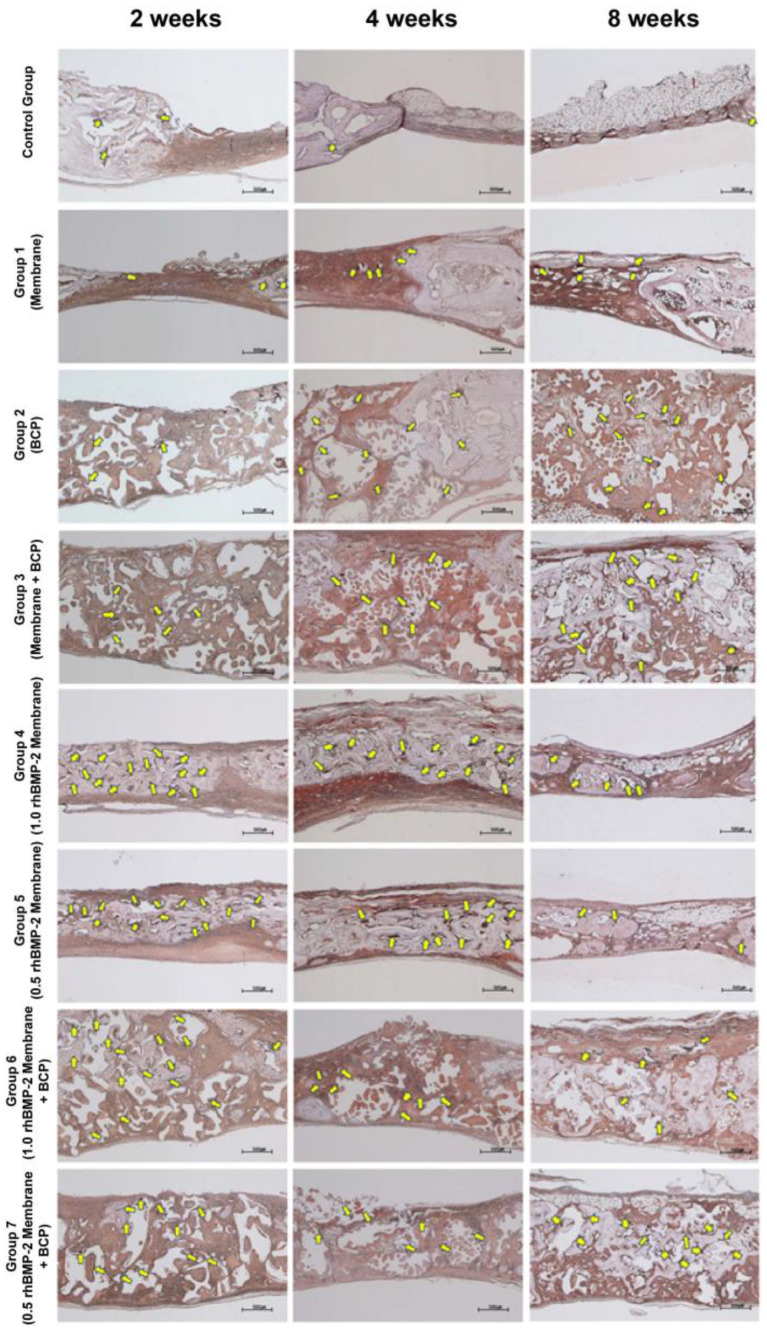
Histological specimens from the control group and group 2 after 2-, 4-, and 8-week healing periods (anti-osteocalcin antibody staining). Yellow arrows indicate osteocalcin as an indicator of cell activity for bone formation. Scale bar: 500 µm.

Figure 7 shows images of OCG3-stained tissue specimens from the control and experimental groups. At 2 weeks, only a small amount of osteocalcin was observed near the defect boundary in the control group and group 1. Osteocalcin was observed around the bone graft material in groups 2, 3, 6, and 7. However, osteocalcin expression was higher in the bone graft material around the existing bone than that in the center of the defect. At 4 weeks, the control group and group 1 showed osteocalcin only in the vicinity of the defect boundary, which was similar to the situation after 2 weeks of healing. At 4 weeks, groups 2 and 3 showed more osteocalcin than in week 2, but there was no significant difference in the defect. Similar to the situation after 2 weeks, many sites expressing osteocalcin were observed near the bone graft material around the existing bone. At 8 weeks, the control group showed osteocalcin localized at the border of the defect in lower amounts than those in the other groups. At 8 weeks, groups 6 and 7, for whom new bone completely filled the upper part of the defect, showed osteocalcin not only below the upper part of the defect but also near the internal bone graft material. At 8 weeks, groups 6 and 7 had higher osteocalcin levels than that at 2 and 4 weeks.

## 4. Discussion

GBR induces bone regeneration in defects by spatially isolating rapidly growing epithelial and connective tissue using membranes that create a favorable environment for bone regeneration. In clinical practice, membranes are used in conjunction with bone graft materials to achieve GBR. The bone graft material is grafted onto large defects to protect the space where bone regeneration can occur, ensure that continuous bone regeneration is possible, and improve the quality of the new bone. BCP, which was used in this study, induced osteoinduction to differentiate mesenchymal stem cells through the surface topography of the graft material [34]. The hydroxyapatite in BCP was degraded by cellular action, such as that of osteoclasts, and β-tricalcium phosphate had a faster chemical dissolution rate than cellular action [35,36]. After grafting, the bone material gradually decreased in volume due to cellular action and chemical fusion and was eventually replaced by new bone. Growth factors are often used to accelerate bone differentiation. Many studies have focused on increasing the regenerative ability to exist bone using bone graft materials and growth factors; however, this study focused on using a membrane acting as a bone wall that could promote stable bone formation. Non-absorbable membranes, such as titanium meshes, have good space retention, whereas absorbable barriers can collapse when applied to large critical bone defects and interfere with bone formation [37,38,39]. The disadvantages of absorbable barriers were supplanted, and bone regeneration was enhanced via the application of rhBMP-2 to a collagen membrane widely used in clinical practice. Critical bone defects are used in bone-regeneration studies because they do not heal naturally and can thus clearly show the effects of the intervention.

In this study, four bone defects with diameters of 8 mm were created in the calvaria of rabbits, and GBR was performed. The rabbits were allowed to heal for 2, 4, or 8 weeks since the rabbit bone remodeling cycle is 6 weeks. The fact that the defects in the control group did not heal completely after 8 weeks suggests that a defect with a diameter of 8 mm is a critical bone defect.

The results of groups 1, 2, and 3 confirm the effect of the presence or absence of a collagen membrane and BCP on bone formation. However, since there was no statistically significant difference in new bone formation among these three groups, it is difficult to confirm the individual effects of the collagen membrane and BCP. However, the lack of SV in groups 2 and 3 could be attributed to space maintenance by BCP. H and E staining showed that the collagen membranes collapsed, and the new bone that formed over the defects was relatively thin in the groups without BCP application. The control group and group 1 were compared with groups 4 and 5 to confirm the effect of rhBMP-2 application on the collagen membrane, which revealed no significant difference in new bone formation. However, the SVs in groups 4 and 5 were smaller than those in the control group and group 1. This means that the new bone rapidly isolated the defect area and enhanced the bone-forming activity in the defect.

Chung et al. used collagen membranes with rhBMP-2 and found that alkaline phosphatase activity increased at 2 weeks, and osteocalcin levels increased 4 days after surgery [31]. rhBMP-2 was stably and continuously released for up to 49 days. The clinical usefulness of rhBMP-2 remains limited because of its low solubility and relatively short biological half-life at a physiological pH [1,40,41,42]. Its application inside the defect can cause excessive adipose tissue formation after healing, which affects the stability of the graft site and the quality of the new bone [25]. Thus, the application of rhBMP-2 to absorbable membranes to rapidly produce new bone that can reinforce them protects the space inside defects. Rapid upper bone formation can protect internal bone regeneration during healing by preventing the invasion of external connective tissue [17,43,44].

The combination of a collagen membrane with rhBMP-2 and BCP in groups 6 and 7 yielded significantly higher bone formation rates than in group 3. Groups 6 and 7 had significantly reduced RB compared to group 3, and new bone was more concentrated in the upper part of the defect. Although there was no significant difference in the bone formation rate between groups 6 and 7, a low concentration of rhBMP-2 is recommended with respect to cost and safety if the same effect can be obtained with 0.5 mL/mg of rhBMP-2. New bone formed differently over time, with no statistically significant difference in new bone formation after 4 and 8 weeks (LSD analysis 2 weeks < 4, 8 weeks).

Pentachrome staining showed immature new bone around the existing bone in all experimental groups after 2 weeks of healing and matured new bone around the existing bone after 4 weeks. After 8 weeks, new bone was observed in the center of the defects, and the boundaries between new and existing bone became blurred in the groups that received collagen membranes with rhBMP-2. In groups 6 and 7, mature new bone connected to the center of the defect became thinner the nearer it was observed to the center of the defect. These results indicate that the collagen membranes with rhBMP-2 maintained space for bone regeneration by causing a bone wall to rapidly grow on the upper part of the defect. However, in groups 1 and 3, which received collagen membranes without rhBMP-2, the new bone did not reach the center of the defects and did not show continuity after 8 weeks.

High osteocalcin levels indicate that osteoblasts are mature and in the process of forming new bone [45]. OCG3 staining showed that osteocalcin in the experimental groups extended from the border to the center of the defect. However, after 8 weeks, differences were observed among the experimental groups. After 8 weeks, in groups 1, 2, and 3, osteocalcin was found up to the center of the defects in relatively high amounts. However, in groups 4, 5, 6, and 7, the levels of osteocalcin decreased, which was not present below the mature new bone generated at the top, although it was present in small amounts around the RB. In the groups that received collagen membranes with rhBMP-2, the rate of new bone formation was initially high but slowed over time because of the rapid release of rhBMP-2. The decrease in osteocalcin levels was lower in groups 6 and 7 than in groups 4 and 5. Thus, the application of BCP and a collagen membrane with rhBMP-2 could reduce the initial burst release of rhBMP-2. In other words, it is likely to cause the new bone formation to gradually increase even after 8 weeks.

This study showed that the application of rhBMP-2 to collagen membranes outside the defect helped reinforce the role of the membranes in the GBR model and confirmed that the combination of a collagen membrane with rhBMP-2 and BCP optimized healing. The application of rhBMP-2 to collagen membranes represents a novel GBR method that rapidly forms a natural bone wall, reinforcing the role of membranes in the defect area. However, this study has three limitations. The first limitation is the relatively small sample size, which could have affected the results for the individual animals. Further studies with larger sample sizes are required to validate these results. The second major limitation is that only two rhBMP-2 concentrations were tested. Further studies with varying concentrations of rhBMP-2 are required to identify the most effective low-dose rhBMP-2 approach. The third major limitation is that the change in the total bone volume was not measured by devising experimental designs that showed the total volume increased or decreased as an absolute value during the healing period. Further research should be conducted to determine the best bone graft material for use with collagen membranes containing rhBMP-2 that can interact with rhBMP-2.

## 5. Conclusions

Collagen membranes with rhBMP-2 form natural bone walls on the upper part of the defects after 4 weeks and can help to form new bone inside the defect even at 8 weeks. This study proposes a novel concept based on GBR, in which rhBMP-2 is applied to collagen membranes outside the defect instead of in the grafted area to rapidly form bone walls early in the healing process, thereby inducing quantitatively and qualitatively enhanced bone regeneration in critical bone defects.

## Figures and Tables

**Table 1:** New bone formation analysis.

Variables	N	New Bone (%)	*p*-Value	Post hoc Analysis with LSD ^a^ Method
Group			<0.001 ***	Control, G1, G2, G3, G4, G5, <G6, G7
Control	10	14.54 ± 4.86		
Membrane (G ^b^ 1)	10	18.50 ± 10.88		
BCP ^c^ (G2)	11	18.31 ± 10.17		
BCP + membrane (G3)	12	22.47 ± 9.02		
Membrane + BMP-2 = 1 (G4)	16	21.58 ± 10.65		
Membrane + BMP-2 = 0.5 (G5)	15	20.30 ± 7.99		
Membrane + BMP-2 = 1 + BCP (G6)	14	31.25 ± 11.62		
Membrane + BMP-2 = 0.5 + BCP (G7)	12	32.56 ± 12.62		
Healing time			0.008 **	2 weeks < 4 weeks, 8 weeks
2 weeks	37	18.71 ± 7.61		
4 weeks	31	23.29 ± 12.34		
8 weeks	32	27.03 ± 12.46		

The values represent the mean ± LSD Statistically significant (**, *p* < 0.01; ***, *p* < 0.001; ANOVA test) Abbreviations: ^a^ LSD, least significant difference; ^b^ G, group; ^c^ BCP, biphasic calcium phosphate.

## Data Availability

All data generated or analyzed during this study are included in this published article.
